# Towards precision medicine: discovering novel gynecological cancer biomarkers and pathways using linked data

**DOI:** 10.1186/s13326-017-0146-9

**Published:** 2017-09-19

**Authors:** Alokkumar Jha, Yasar Khan, Muntazir Mehdi, Md Rezaul Karim, Qaiser Mehmood, Achille Zappa, Dietrich Rebholz-Schuhmann, Ratnesh Sahay

**Affiliations:** Insight Centre for Data Analytics, NUIG, Galway, Ireland

**Keywords:** Cancer genomics, Biomarkers, Multi-Omics, Pathways, Gynecological cancer, Linked data, Semantic technologies

## Abstract

**Background:**

Next Generation Sequencing (NGS) is playing a key role in therapeutic decision making for the cancer prognosis and treatment. The NGS technologies are producing a massive amount of sequencing datasets. Often, these datasets are published from the isolated and different sequencing facilities. Consequently, the process of sharing and aggregating multisite sequencing datasets are thwarted by issues such as the need to discover relevant data from different sources, built scalable repositories, the automation of data linkage, the volume of the data, efficient querying mechanism, and information rich intuitive visualisation.

**Results:**

We present an approach to link and query different sequencing datasets (TCGA, COSMIC, REACTOME, KEGG and GO) to indicate risks for four cancer types – Ovarian Serous Cystadenocarcinoma (OV), Uterine Corpus Endometrial Carcinoma (UCEC), Uterine Carcinosarcoma (UCS), Cervical Squamous Cell Carcinoma and Endocervical Adenocarcinoma (CESC) – covering the 16 healthy tissue-specific genes from Illumina Human Body Map 2.0. The differentially expressed genes from Illumina Human Body Map 2.0 are analysed together with the gene expressions reported in COSMIC and TCGA repositories leading to the discover of potential biomarkers for a tissue-specific cancer.

**Conclusion:**

We analyse the tissue expression of genes, copy number variation (CNV), somatic mutation, and promoter methylation to identify associated pathways and find novel biomarkers. We discovered twenty (20) mutated genes and three (3) potential pathways causing promoter changes in different gynaecological cancer types. We propose a data-interlinked platform called BIOOPENER that glues together heterogeneous cancer and biomedical repositories. The key approach is to find correspondences (or data links) among genetic, cellular and molecular features across isolated cancer datasets giving insight into cancer progression from normal to diseased tissues. The proposed BIOOPENER platform enriches mutations by filling in missing links from TCGA, COSMIC, REACTOME, KEGG and GO datasets and provides an interlinking mechanism to understand cancer progression from normal to diseased tissues with pathway components, which in turn helped to map mutations, associated phenotypes, pathways, and mechanism.

## Background

Next Generation Sequencing (NGS) technologies open new diagnostic and therapeutic ways for cancer research. The resulting high-throughput sequencing data has to be processed in complex data analytics pipelines including annotation services. Unfortunately, there is not yet a well-integrated platform available for both clinical and translational [1–5] research to fulfill these annotation and analytical tasks. In addition, the large volumes and growing variety of NGS data sources pose another challenge, since the computational infrastructure for the biological interpretation will have to cope with very large quantities and heterogeneities of data originating from sequencing facilities [6–8]. More importantly, the functional annotation of genomics data for cancer has to take tissue-specificity into consideration and thus has to avoid ambiguity while consolidating and aggregating clinical outcomes from disparate resources. Similarly, a computational platform that can consolidate variety of data derived from electronic health records (EHRs), omics technologies, imaging, and mobile health is a fundamental requirement to accelerate the recent precision medicine initiative^1^ [9]. In our initial work [10] we presented an approach to link and query three large repositories – TCGA^2^, COSMIC^3^, and Illumina Human Body Map 2.0^4^ – to analyse the expression of specific genes in different tissues and its variants by:

- Linking of gene expression, copy number variation (CNV), somatic mutation data from two disjoint resources (i.e., COSMIC and TCGA).
- Identifying sets of genes using the Illumina Human Body Map 2.0 with relevance for ovarian cancer with a comprehensive set of mutations.

In order to analyse the tumorigenesis of female gynecological cancer types, in this article we extend our previous work [10] by including:

- Ovarian Serous Cystadenocarcinoma (OV), Uterine Corpus Endometrial Carcinoma (UCEC), Uterine Carcinosarcoma (UCS), Cervical Squamous Cell Carcinoma and Endocervical Adenocarcinoma (CESC) datasets.
- Methylation data to further understand potential promoter genes based on methylation change and biomarkers.
- REACTOME, KEGG and GO biological processes datasets to understand cancer causing gene regulation through associated pathways and biological processes.

To further understand the epigenetics, we retrieved the genomic positions (loci), mutation frequency, change in promotor methylation for each gene in the above four cancer types (OV, UCS, UCEC, & CESC). These are further classified by biological processes involved in understanding the mechanism and associated pathways. By doing this we explore the variant and mutation prioritization using 16 different tissue types reported in the Illumina Body Map 2.0. The differential expressed genes derived from Illumina Human Body Map 2.0 – using the procedure suggested by Trapnell, C. et al. [11] – are linked with different tissue types and gene expressions in COSMIC and TCGA datasets leading to a potential biomarker for a particular tissue-specific cancer.

The proposed approach enriches mutations and methylation by filling in missing links from COSMIC, TCGA, REACTOME, KEGG and GO datasets providing a mechanism to analyse cancer progression from normal to diseased tissues with key pathway components. Our key objective is to understand the tumorigenesis of these four gynecological cancer types (OV, UCS, UCEC, & CESC). In order to retrieve the patterns of genes and tissue-specific information from various cancer mutations reported in multiple repositories; we encountered three computational challenges for linking and querying these multiple distributed repositories: (i) transform heterogeneous data repositories and their storage formats into standard RDF; (ii) discovering links by finding specific patterns, i.e., correlations for a gene with regards to CNV, mutation, gene expression, and methylation datasets; and (iii) scalable querying over the large volume datasets covering 16 different tissue types and the gene expression data from different repositories. We propose a data-interlinked platform called BIOOPENER^5^ that enables automated discovery of data linkages and querying of information from large-scale cancer and biomedical repositories.

The experiments conducted in this paper is aligned to the transcriptome and epigenetics studies based on the Human Body Map 2.0 (HBM) from Illumina which covers the following tissues: adrenal, adipose, brain, breast, colon, heart, kidney, liver, lung, lymph, ovary, prostate, skeletal muscle, testes, thyroid, and white blood cells. The HBM provides gene-specific information across one or more tissue types and intends to support the identification of potential biomarkers for a targeted therapy. In this study, our results not only discover novel biological outcomes but also provides a linked datasets that assimilates clinical outcomes from related data repositories.

The rest of the paper is structured as follows: “Motivation” section motivates our working scenario based on Illumina Human Body Map (HBM) 2.0, cancer and biomedical databases (COSMIC, TCGA, REACTOME, KEGG and GO); “Methods” section presents the BIOOPENER methodology and architecture; “Results” section discusses the results obtained from the BIOOPENER platform; “Related work” section presents the related work in linking and querying cancer genomics repositories; and “Conclusion” section draws the conclusion from our work.

## Motivation

In order to understand the tumorigenesis, it is one approach to compare normal and diseased tissue samples to interpret the changes in the expression patterns of the genes with regards to the observed disease status. In our case, Illumina Human Body Map (HBM) 2.0 serves the purpose to identify similarities in gene expression patterns using the studies across different tissue types, where HBM discloses the similarities between human tissues on the molecular and genetic level. Due to overlaps between cancer behaviors, progression, and mutated genes, we have selected top 100^6^ genes by a filtering criteria based on the Reads Per Kilobase of transcript per Million mapped reads (RPKM) values. Further, these top 100 genes identified are linked using the genetic features such as genomic loci (start, end), beta value, cell cycle etc. from previously observed studies in COSMIC and TCGA repositories. The work presented in this article covers only non-synonymous (NS) mutations. Since many somatic mutations are passenger – synonymous mutations – and do not impact tumorigenesis, we first select those genes that are more likely to be drivers. The selection of driver genes is based on the mutations frequency (RPKM value).

**Illumina Human Body Map (HBM) 2.0:** HBM covers data from transcriptome studies for 16 tissue types. Samples for these 16 tissue types have been processed, aligned and finally expression level have been determined [12]. Sequencing has been performed to provide both paired-end and single-end libraries (read-length of 50bp and 75bp). A list of differentially expressed genes are extracted using the step 2 (assemble expressed genes and transcripts) of procedure suggested by Trapnell, C. et al. [11]. The gene expression data extracted from HBM samples returns a very large list of more than 52000 genes. For data processing reasons we chose to reduce the list and therefore defined the cut-off for each *RPKM* value according to the method suggested by Sandberg et.al [13]. As a result, the data for each tissue type includes both the coverages and the *RPKM* values as the corresponding expression level. The RNA seq dataset provides additional relevant data such as CNV, fusion genes, structural variation, differentially expressed genes, novel mutations, splice junctions and transcriptome variations [14].

**Fig. 1:**
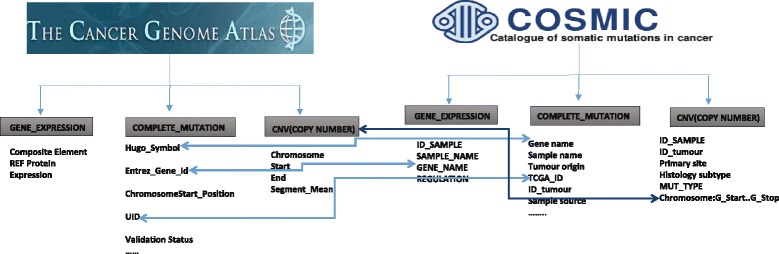
Links between COSMIC and TCGA datasets

**Annotation Databases (COSMIC & TCGA):** he main focus of this work is the identification of patterns for cancer mutations and globally known mutations and their types for selected differentially expressed genes across different tissue types. Figure 1 shows the correspondences, i.e., the associations or links that have been established between the TCGA and COSMIC databases for this purpose. For this task, our primary concern has been the associations between the CNV, the known mutations, and the gene expression data.

As part of our initial work [10], we have identified instances to link in the COSMIC and TCGA datasets (see Fig. 1). For example, GENE_NAME is used to establish links between COMPLETE_MUTATION and GENE_EXPRESSION datasets between both the repositories. Similarly, GENE_NAME and HUGO_SYMBOL has been used to link COMPLETE_MUTATION from both the datasets. Further, CNV datasets from COSMIC and TCGA have been linked based on chr:start_end position. From the computational perspective, the links (*owl:sameAs*) between COMPLETE_MUTATION and GENE_EXPRESSION datasets using the GENE_NAME property allow to create a subset of driver genes from a larger complete set of mutations.

**Annotation Databases (REACTOME, KEGG, & GO processes):** We observe a set of prospective links through the DNA methylation datasets – from COSMIC and TCGA – to GO proliferation Ids. These links broaden our understanding of the cell proliferation (with frequently mutated genes) where changes in methylation level regulate the gene expression. In order to target certain genes, it is important to find the affected cancer types and the common pathways associated with the cell proliferation. The KEGG and REACTOME datasets provide additional links to identify genetic profiles from already identified mutations in COSMIC and TCGA datasets. Clinical variations of any mutation from the REACTOME dataset will help to explore clinical relevant targets, effects of down-regulation of each pathway and alternate pathways for the cell.

**Fig. 2:**
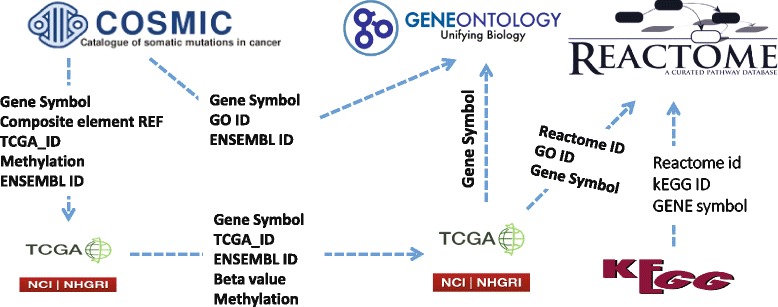
Links between COSMIC, TCGA, REACTOME, KEGG, and GO datasets

Figure 2 shows a set of prospective *owl:sameAs* links between COSMIC, TCGA, REACTOME, KEGG, GO datasets. For example: (i) if “Gene Symbol” used in the TCGA gene expression gets linked (through *owl:sameAs*) with the “Gene Symbol” of COSMIC methylation datasets, then a simple query can fetch result about the changes in a promotor region associated with mutations already identified in TCGA and COSMIC datasets; (ii) similarly, “ENSEMBL ID” used in COMSIC, TCGA, and Gene Ontology datasets can be linked to obtain the transcript level changes with mutated gene in order to understand the disease progression; (iii) finally, by linking COSMIC and TCGA “Methylation” datasets provides us the measure of beta value changes, the responders, and non-responders based on hyper and hypomethylation. In our initial work [10], we have identified MYH7 as one of the potential biomarker based on copy number variation (CNV) frequencies. In this article, we are aiming to link the identified mutations (from COSMIC & TCGA) across KEGG, REACTOME, and GO datasets to understand the metabolic process of each reaction and the localization of each component of a reaction further connecting the metabolic process to pathways described in the KEGG dataset.

## Methods

The BIOOPENER approach is fundamentality similar to the Bio2RDF^7^[15, 16] framework that created a mashup of linked data connected through various linking properties (e.g., *xRef*, *owl:sameAs*, *x-relation*) [17]. BIOOPENER focus is specifically around discovering and exploiting the *owl:sameAs* links for constructing complex federated queries – due to the precise *owl:sameAs* semantics [18] – across multiple datasets. We now present the BIOOPENER’s architectural, linking, and querying methodology.

### BIOOPENER architecture

**Fig. 3:**
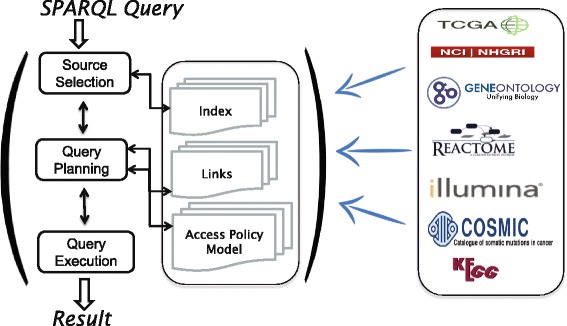
BIOOPENER: Linking & Querying Cancer Genomic Resources

The BIOOPENER architecture is summarized in Fig. 3 showing all three major components. First, the RDFization component that generates Linked Data from the COSMIC, TCGA, REACTOME, KEGG, GO databases results into several SPARQL endpoints. It is important to note that, the two datasets (COSMIC and TCGA) are converted from the raw format to RDF; further, we linked COSMIC and TCGA to REACTOME^8^, KEGG^9^, and GO^10^ datasets hosted at the Bio2RDF^11^. Second, the linking component searches and discovers links between selected datasets. The links discovered by this component have an effect on the efficiency of the source selection, on the query planning, and on the overall query execution over distributed SPARQL endpoints. Third, the scalable query federation component: it a single-point-of-access through which distributed data sources can be queried in the concerto.

The scalable query federation is based on the SPARQL query federation engine called SAFE [19], which has been developed for accessing distributed clinical trial repositories. SAFE provides a single-point-of-access through which distributed data sources can be queried in unison. SAFE has been adapted to improve the efficient integration of data from the different COSMIC, TCGA, REACTOME, KEGG, GO SPARQL endpoints. More specifically, SAFE makes use of a favorable distribution of data to reduce the number of sources required for processing federated SPARQL queries (without compromising recall). SAFE retrieves results from the large-scale repositories by (i) efficient source selection as per the capabilities of genomics repositories; (ii) query planning mechanism to decompose a query and build resultant data set from several sub-queries; (iii) query optimisation to execute the sub-queries; and (iv) query execution mechanism retrieve and integrate results. This approach is based on the principle that integrated data sources allow querying of multiple data sources in a single search, independently of their status being distributed or centralized, whereas traditional methods of data integration rather map the data models to a single unified model.

### RDFization

The raw data files – of COSMIC and TCGA repositories – are available in the tab separated text (tsv) format, which are transformed into the RDF format using our in-house RDFizer tool that generates the N3 triples. The transformed RDF data from each cancer type are hosted as different SPARQL endpoints. The four types of data have been included from COSMIC, i.e., gene expression, gene mutation, CNV, and methylation. From TCGA we have RDFized three types of data, i.e., CNV, gene expression and methylation for four cancer types, namely *Ovarian Serous Cystadenocarcinoma* (OV), *Cervical Squamous Cell Carcinoma and Endocervical Adenocarcinoma* (CESC), *Uterine Corpus Endometrioid Carcinoma* (UCEC) and *Uterine Carcinosarcoma* (UCS). Table 1 shows the overall statistics of the RDF datasets: row 1 represents for the COSMIC gene expression data the corresponding triples generated (column 3), the number of subjects (column 4), the number of predicates (column 5), the number of objects (column 6) and it’s RDF data size (column 7). Rows 2-4 represent the same type of data for the COSMIC gene mutation, CNV and methylation data, respectively. A total of 154 million records has been RDFized, producing approximately 1.2 billion triples, for COSMIC datasets. Row 5-8 represents the statistics for the RDF version of TCGA-OV, TCGA-CESC, TCGA-UCEC, and TCGA-UCS, respectively. Rows 9-10 represent the RDF data statistics for KEGG, REACTOME and GOA datasets, respectively. These three datasets are external as we have not transformed them into the RDF format but instead used the already available RDF versions from Bio2RDF.

**Table 1:** RDF Data Statistics

No.	Data	Triples	Subjects	Predicates	Objects	Size (MB)
1	COSMIC GE	1184971624	148121454	18	148240680	10000
2	COSMIC GM	83275111	3620658	23	9004153	1400
3	COSMIC CNV	8633104	863332	10	921690	122
4	COSMIC Methylation	170300300	8292057	22	603135	2800
5	TCGA-OV	81188714	10974200	15	4774584	3774
6	TCGA-CESC	3763470	627652	43	481227	49557
7	TCGA-UCEC	553271744	19233824	91	68370614	84687
8	TCGA-UCS	1120873	183602	36	188970	10018
9	KEGG	50197150	6533307	141	6792319	4302
10	REACTOME	12471494	2465218	237	4218300	957
11	GOA	28058541	5950074	36	6575678	5858

### Linking

We propose a linked data based approach to create correspondences (links) between dispersed cancer and biomedical datasets. These datasets contain rich information and helpful in answering the biological questions targeted in this article. These links, once identified and established, will sustain and support the query federation over distributed repositories (discussed in the “Scalable query federation” section).

**COSMIC and TCGA linking:** we perform linking of the COSMIC and TCGA datasets. We have employed the owl:sameAs construct to establish links across entities based on the semantic properties highlighted in Fig. 1. For example, the entities that contain information about *Gene Symbol, TCGA_ID, ENSEMBL ID* have been linked using owl:sameAs. An example link between COSMIC and TCGA is shown in the Listing 1, where two COSMIC sample ids have been identified as being identical to two TCGA patient bar code ids.



**Fig. 4:**
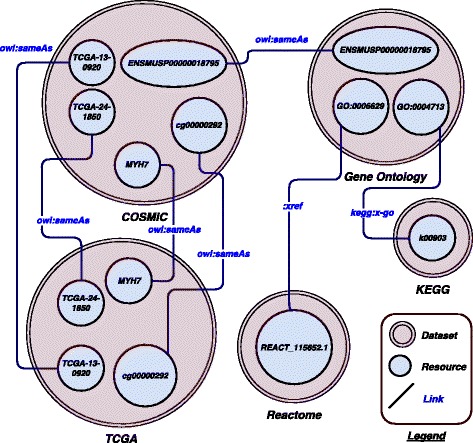
Example Links between COSMIC, TCGA, KEGG, REACTOME, and GO Datasets

The example links generated in our use-case are shown in the Fig. 4. The COSMIC and TCGA datasets have been integrated using the owl:sameAs construct. For instance, MYH7 (which is an RDF resource of type Gene Symbol) in both COSMIC and TCGA datasets is linked using owl:sameAs. To understand the promotor genes and their deviation, the methylation datasets of COSMIC and TCGA are linked to retrieve beta values for a given set of CNVs. For instance, *cg00000292* which is an RDF resource of type “Composite Element REF” in both COSMIC and TCGA datasets have been linked using owl:sameAs. Similarly, Fig. 4 shows the owl:sameAs links between COSMIC and TCGA datasets for TCGA-13-0920 and TCGA-24-1850 (RDF resources of type Sample_ID).

**Linking COSMIC and TCGA with REACTOME, KEGG, & GO:** We link COSMIC and TCGA with Gene Ontology (GO) datasets to understand the biological processed involved with each mutation or CNVs and the underlying impact of these mutations on cancer and healthy cells. From the Fig. 4, it is evident that we have linked *ENSMUSP00000018795* – which is an RDF resource of type Ensemble ID – in COSMIC dataset with the similar resource in GO dataset. This will help in retrieving the gene behavior of healthy cells (from Illumina Body Map) compared to the diseased TCGA samples by tracking the GO process involved in the oncogenesis. By enabling links between COSMIC and GO datasets, we are now able to find links across Reactome and KEGG datasets. This will allow tracking the changes in healthy cells based on their pathway activities to identify the disease and biological process related pathways. For instance, the “Ensemble ID” from COSMIC is linked with the “Ensemble ID” in GO dataset providing us the GO processes and the GO IDs associated with these processes. These are further linked with their respective KEGG and Reactome IDs. The linking across these datasets are shown in Fig. 4.

**Fig. 5:**
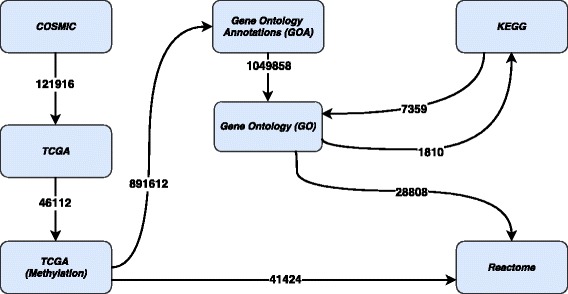
Link Statistics

The number of links generated in case of COSMIC and TCGA datasets, and the number of identified links between KEGG, GO, and Reactome datasets are shown in the Fig. 5. For instance, a total number of 121916 links are generated in COSMIC to link them with TCGA. Similarly, 46112 links are generated to integrate TCGA with TCGA Methylation datasets, 891612 links are generated to link TCGA Methylation dataset with GOA (Gene Ontology Annotation) dataset, and 41424 links are generated to integrate TCGA Methylation dataset with Reactome.

On the other hand, we identified a total of 1049858 existing links – within Bio2RDF – between GOA and GO datasets. A total of 1810 outgoing links to KEGG from GO and 7359 incoming links to GO from KEGG were identified. A total of 28808 links were discovered between GO and Reactome datasets.

### Scalable query federation

We have developed a query federation engine – called SAFE — for accessing sensitive clinical data at different locations [19]. Two main changes have been introduced to SAFE for efficiently querying the COSMIC, TCGA, KEGG, Reactome, and GO SPARQL endpoints. First, standardise RDF query representation: in the initial version [19], SAFE issues queries for statistical clinical information stored within distinct names graphs for RDF data cubes [20]. Therefore, the internal query processing (i.e., source selection, query planning, query execution) had to be adapted to query the regular RDFized versions of the COSMIC, TCGA, KEGG, Reactome, and GO datasets. Second, access control had to be disabled: SAFE imposes restrictions for data-access as a feature (defined as Access Policy Model [19]) while federating queries over multiple clinical sites, i.e., imposing the data restrictions for different data repositories. Since experiments conducted in this paper mainly involve public repositories this feature has been disabled.

Figure 3 shows SAFE’s three main components within the BIOOPENER platform: (i) Source Selection: performs multilevel source selection based on the capabilities of data sources; (ii) Query Planning: filters the selected data sources based on access rights defined for each user; and (ii) Query Execution: performs the execution of sub-queries against the selected sources and merges the results returned.

**Fig. 6:**
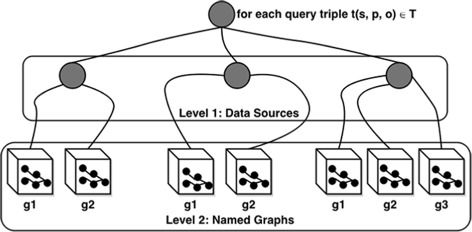
Tree-based two level source selection

**Source Selection:** SAFE performs a tree-based two-level source selection as shown in Fig. 6. At Level 1, like other query federation engines [21–23], we do *triple-pattern-wise endpoint selection*, i.e., we identify the set of relevant endpoints that will return non-empty results for the individual triple pattern in a query. At Level 2 (unlike other query federation engines), SAFE performs *triple-pattern-wise named graph selection*, i.e., we identify a set of relevant named graphs for all relevant endpoints already identified at Level 1. SAFE relies on data summaries to identify relevant named graphs.

**Query Execution:** The Listing 2 shows an SPARQL query, which federates across COSMIC and TCGA data asking for genomic *loci* of a mutated gene by chromosome start points which then returns the disease metastasis information along with the mutation type. Answering such a query requires the integration of COSMIC with TCGA and merging results from both TCGA and COSMIC, and thus has to make use of query federation. The results for the first four triple patterns in the given query (i.e., cosmic:sample, cosmic:gene, cosmic:start) are fetched from COSMIC and the results for the next four triple patterns (i.e., tcga:hybrid_ref, tcga:gene, tcga:start) are fetched from TCGA. Further, both results are merged on the basis of the last triple pattern (gene_c owl:sameAs gene_t) which integrates COSMIC with TCGA. Sample results for this query can be seen in Fig. 9.



In our initial work [10] we queried mutations and CNV data to identify the novel mutations and their somatic behavior from healthy to cancer cells. The Listing 3 shows a SPARQL query, which extracts promoter level changes occurred due to mutations extracted from query shown in the Listing 2. This requires linking across the COSMIC and TCGA Methylation datasets. The first three triple patterns fetch data from COSMIC and the next three triple patterns fetch data from TCGA. The last triple pattern provides a link – *owl:sameAs* between genes – for merging data from both the data sources.



The SPARQL query listed in Listing 4 have covered 3 distinct sources, i.e., methylation from TCGA and COSMIC datasets with associated Gene Ontology Annotations (GOA). TCGA provides the changes in methylation per composite element, whereas in COSMIC we have such changes on the gene level. To retrieve both the gene and promoter level information, we have queried genes from both data sources and extracted all the promoter regions. Once the promoter regions are identified, it is essential to understand the processes involved in these regions. This helped us to query GOA for extracting the processes on the promoter and gene levels. If a gene level change do not comply with promoter level changes, it is an indication of what processes of the gene have mutated them. Such results can be obtained through a federated query with three data sources, i.e. COSMIC, TCGA, and GOA. The Listing 4 provides an example federated query where the first three triple patterns are answered from COSMIC, the next three triple patterns are answered from TCGA and the seventh triple pattern merges result obtained from COSMIC and TCGA through gene. The eighth and ninth triple patterns fetch data from GOA which is finally merged with COSMIC and TCGA datasets using the gene information.



The SPARQL query shown in Listing 5 finds associations between the genes, pathways and biological processes. We queried the healthy genes from Illumina Body Map against all mutations obtained from TCGA and COSMIC to find their DNA and promoter level methylation changes. In order to explore the gain and loss on a disease at the phenotype level, we have included KEGG and REACTOME sources which map each discovered gene with its biological process for phenotype and process driven pathways. The Listing 5 shows a federated SPARQL query, where the first three triple patterns are answered from TCGA; and the next five triple patterns fetch and merge data from REACTOME and GOA. The last five triple patterns obtain results from KEGG and merge them with the rest of results.



The Listing 6 retrieves the methylated promotor regions. The query shown in Listing 6 extracts the location of methylation based on the input genes, composite element REF (promotor region) and chromosome number. For instance, we have queried MYH7 (gene) for promotor region cg05744229 at the chromosome 14 (region of methylation) and extracted two promotor regions from TCGA and COSMIC with the start value of DNA promotor range such as 23904678 (TCGA) and 23435469 (COSMIC).



Listing 7 shows an example federated SPARQL query derived from the Listing 2 for a specific gene, namely MYH7. Similarly, we have executed the federated queries shown in the Listings [2-6] for each of the hundred (100) genes extracted from the Illumina Body Map, mentioned above.



The query execution time for these gene-specific queries is shown in the Table 2. The “Query” column lists individual queries (e.g., listings [2-6]), “QE Time”, “Results (No. of Triples)” and “Datasets” columns show the query execution time in millisecond (msec), number of triples returned as a result and the datasets required for executing individual queries.

**Table 2:** Query Execution Time (**QE**=Query Execution)

Query	QE Time (msec)	Results (No. of Triples)	Datasets
Listing 2	2110	21390	(TCGA)(COSMIC)
Listing 3	5732	33264	(TCGA)(COSMIC)
Listing 4	43092	63765	(TCGA)(COSMIC)(GOA)
Listing 5	263463	232848	(TCGA)(GOA)(REACTOME)(KEGG)
Listing 6	3481	25669	(TCGA)(COSMIC)

## Results

**Fig. 7:**
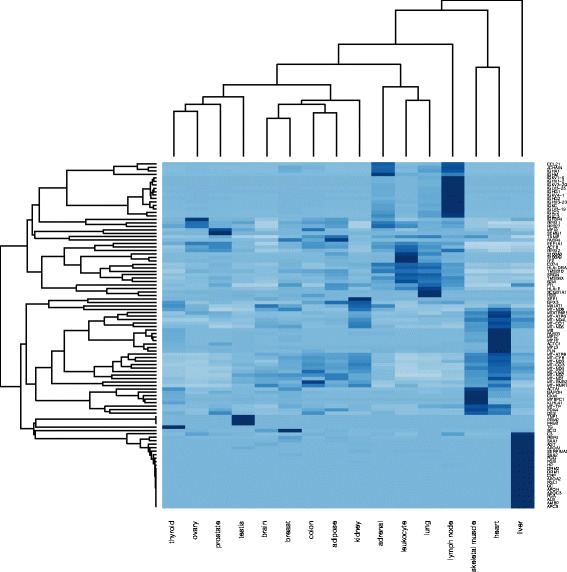
HBM: List of genes expressed in all tissues and highly expressed
- Analysing the normal tissues expression levels against the somatic mutations linked and retrieved from the COSMIC and TCGA datasets.
- Classifying the mutations obtained from above two steps in terms of biological processed and pathways from GO, KEGG, and REACTOME

We analyse the genes having RPKM value > 0.3747 and differentially expressed in all tissue types. Figure 7 shows a list of 100 genes retrieved from the HBM datasets, which are highly expressed in 16 different tissues. We have identified potential cancer types based on the gene patterns for different tissues that helped further to understand the behavior of most amplified cancer types. The overall goal of this study is to understand the relevance and association of mutation, genes expression, and promoter region by:

- Analysing the normal tissues expression levels, enriched and affected pathways along with their associated expression levels and changes obtained from the HBM 2.0 datasets.

We now discuss and analyse the results obtained from the BIOOPENER platform through linking and querying the cancer and biomedical repositories.

### Analysis: HBM, COSMIC, and TCGA

**Fig. 8:**
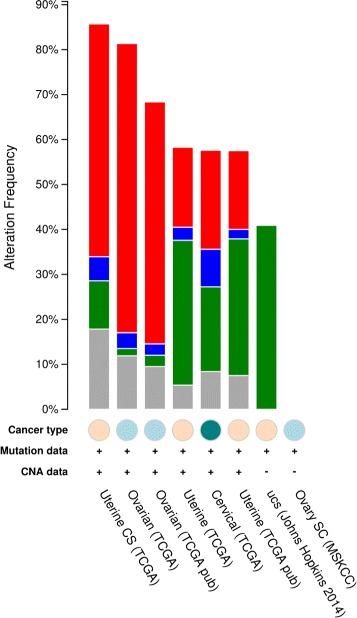
TCGA query output from cBIO Portal (Blue:Deletion, Red:Amplification, Green:Mutation, Brown:Multiple Alterations) [43]

Initially, we have selected top 100 genes that are highly expressed in all 16 tissues as shown in the Fig. 7 to (i) retrieve their CNV, mutation, gene expression and methylation annotations from cBioPortal^12^; (ii) retrieve methylation from CNV annotator^13^ and UCSC Cancer Genomics Browser^14^; and (iii) retrieve mutation datasets from TCGA [24]. The results from TCGA (Fig. 8) clearly indicate a mutation frequency elevated distribution of these genes in *UCS*, *CESC*, *UCEC* and *OV* cancers. In Fig. 8 we observe average percentage case mutations in the UCS, UCEC, CESC and OV cancers are 87.5%,58.3%, 57.6%, 81.4% respectively. This outcome justifies the selection of UCS, UCEC, CESC and OV as good candidates for further investigation due to its elevated amplification rate and its multiple repetition in different experiments.

**Fig. 9:**
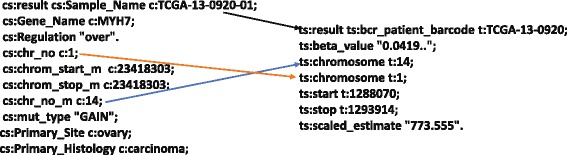
Linked annotations for MYH7 - COSMIC

This study targets genes based on their contribution in mutations^15^, the listing 8 shows the highly relevant driver genes transforming healthy human tissues into diseased ones for respective cancer types.



The overlap and frequency among these four cancer types results into the discovery of top 20 biomarkers shown in the listing 9). Table 3 shows the potential chromosome locations ***chr14,chr5,chr6,chr19*** and genes ***TG,TXNIP,GC,MYH7*** with high relevance in the progression of four gynecological cancer types.

**Table 3:** loci information for highly expressed gene in ovarian cancer from HBM 2.0

Chr	Star-End	Mutation Type	Genes	PMID
19	90910 -715430	GAIN	FGF22, RNF126, TG	2066845 120668450
9	4069657-4684967 591967-608659 11090336-11098891 8009428-8015596 8109010-8121257 1373387-1383725 11090336-11098891 10547511-10547923 3113846 -3134738 8115293 -8121487 9269903 -9294415 46587-510700 5106680-5106800	LOSS/GAIN	LKB1,P16INK4A,TRAF2,XPA, PTCH1,FANCC,DMRT3,WNK2,C9orf89, SYK,CKS2,CTSL1,NTRK2,KIF27,PTPRD, TLE4,CEP78,GNAQ,PRKACG	21062161 17311676 1658517020668451 21781307
6	149661-384546	LOSS	TAP1,NOL7,CD83,POUF3,MYH7,PLN,PKIB,PDSS2 OSTM1,NUS1,TG,NT5DC1,NR2E1,NKAIN2	21062161 20668451 21781307 20668451 21720365
5	15532-24132	GAIN	TRIP13, TRIO,TARS,SUB1,SLC12A7, SKP2,SDHA,RPL37,MYH7,RNASEN,RAI14, RAD1,POLS,PDCD6,PAIP1,OSMR,NNT	18559093 21062161
14	23857092-23886486 23857082-23886607	LOSS	MYH6, MYH7, TG, ACTA1	18559093 21062161



Figure 9 shows the COSMIC and TCGA annotations. The CNV datasets doesn’t use “Gene symbol” property (or predicate) and it is important to map (or link) genome regions with gene symbols to retrieve CNV information from different datasets. We implemented a linking rule based on the *chr_no,chr_start and char_end* properties (or predicates) to retrieve the CNV information across datasets to identify genes within the extracted *loci*. Result of this annotation are shown in the Table 3. It is evident that the MYH7 gene has many copies reported in the COSMIC datasets as well as in the TCGA datasets suggesting it a potential biomarker for four gynecological cancer types. The TG and MYH7 genes are highly mutated as they are repetitively appearing on multiple chromosomes. For instance, *MYH7* primarily carried the LOSS type of a mutation for *chr14* which is a dominant mutation with all its regulation of over, under and normally expressed. Translational researchers may want to repeat and re-validate the study for Pubmed ID:1398522 with the *beta value* – as a measure of methylation – of 0.041999536. The scaled estimation (Tumour purity) of 773.555 supports this gene (*MYH7*) from the methylation aspect to detect promoter level changes in the four cancer types. Further multiple genomic locations will help clinical practitioners to find a potential CNV for a targeted study ultimately helping towards a better prognosis.

**Fig. 10:**
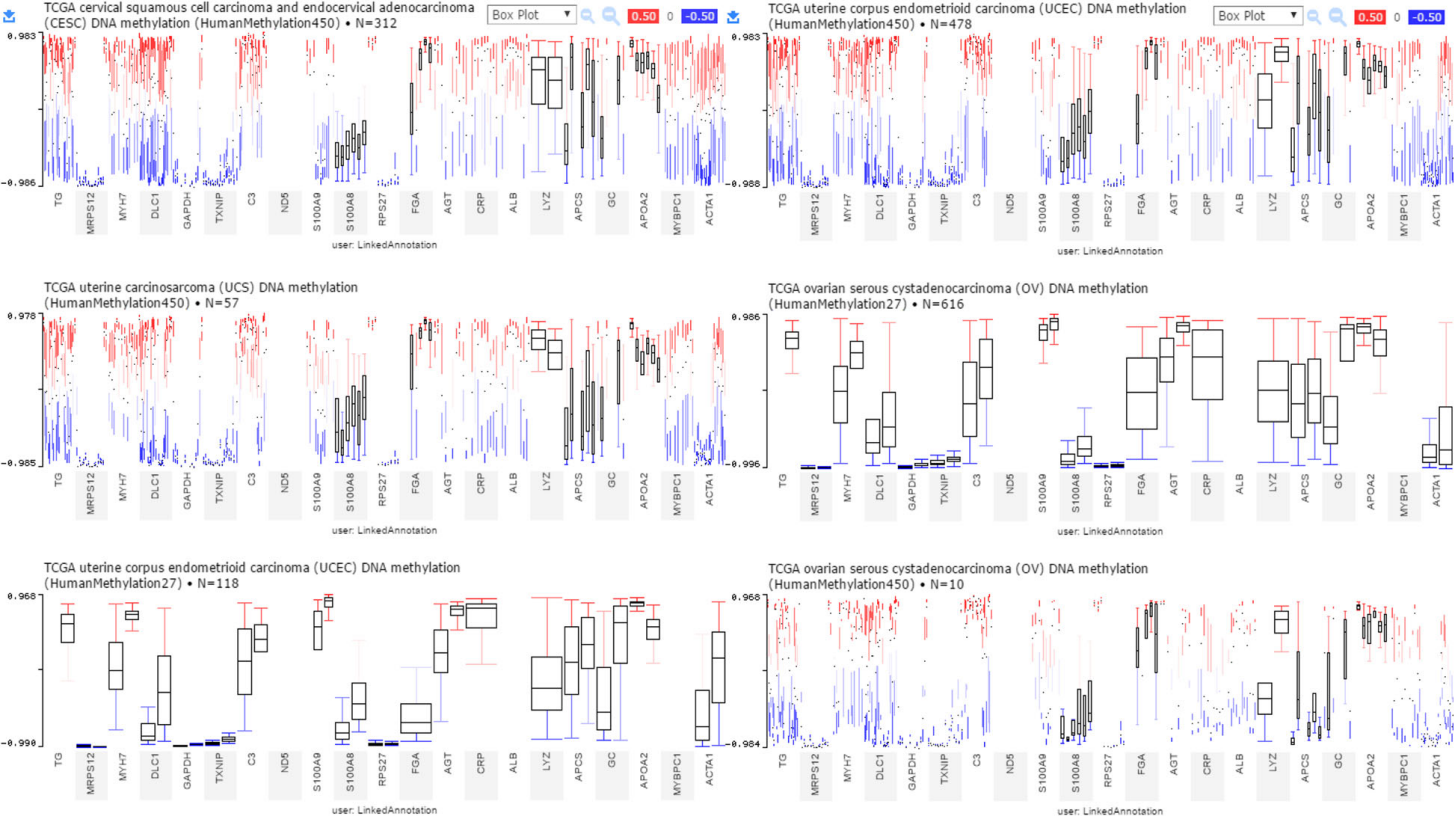
Promotor level methylation changes in biomaker genes

Figure 10 shows the annotation of twenty (20) discovered biomarkers (genes) where promoter level changes are occurring on the extreme changes of -ve or +ve beta-values in all the four cancer types studied in this article. The most affected genes due to these promotor level changes are: **MYH7, TG, DLC1, S100A8**. As reported in our initial work [10] major changes are occurring nearby -0.773 beta-value and their corresponding composite element reference ids are *cg01429391, cg05744229, cg26670875, cg18205205, cg21242212, cg08240074, cg13785779, cg05744229*. Most of these changes are occurring around chromosome 1 and 14 and 5’ UTR. Next section discusses the mechanism behind these changes and their pathway analysis.

### Analysis: GO, KEGG and REACTOME

**Fig. 11:**
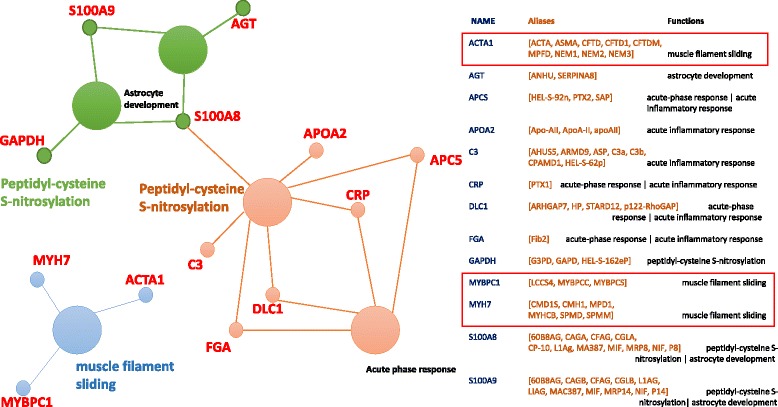
Three pathways causing promoter changes in four gynecological cancer types

We have identified twenty (20) genes in terms of mutation frequencies and CNV together with the promoter level changes in methylation data. However, we are unaware of the mechanism involved in combined effects of these twenty (20) genes. We have queried linked pathways and coalitions over the GO, KEGG, and REACTOME datasets. Figure 11 – snippet generated from ClueGO [25, 26] – shows the *muscle filament sliding* pathway as a key in rare cancer types such as retinoblastoma where effective “actin” filament formation with Myosin (MYH4) is a prime regulator [27]. Our approach has identified “actin (ACTA1)” and “myosin (MYH7)” combination with “MYBPC1” as the potential pathways causing promoter changes in gynecological cancers. It’s evident that alterations in the activity and/or expression patterns of actin-bundling proteins could be linked to the cancer initiation or progression [28]. Haitian Lu, et al. suggests that the *acute inflammatory response* is associated with cancer development because inflammatory micro-environment inhabits various inflammatory cells [29]. A network of signaling molecules are indispensable for the malignant progression transformed cells attributed to the mutagenic predisposition of persistent infection-fighting agents at the sites of chronic inflammation causing cancer development in various tissues [29]. In our case, the reason behind significant methylation changes associates with the pathway *peptidyl-cysteine s-nitrosylation*. The dysregulation of s-nitrosylation in severe pathological events including cancer onset, progression, and treatment resistance leads to controlled epigenetic and treatment response [30]. Figure 10 explains the gene associated with each pathway and their contribution for OV, CESC, UCS, and UCSC cancer types. In this article, we demonstrated that well-connected datasets allow to construct complex biomedical queries (e.g., listings 2-6) covering variety of genetic and biological features (cnv, gene symbol, methylation, cell cycle, protein, pathway, etc.) that can span through broad range of multiple repositories.

## Related work

Kandoth et al. [31] performed a cancer study with 12 cancer types to enable logical classifications for the large amount of data generated by TCGA and ICGC. Saleem et. al. [32] have covered TCGA database with few cancer types and for a limited number of patient data. Similarly, a reduced version of the COSMIC database has been RDFized to explore on the mechanism of TP53 [33]. The federation platform [34] called “TopFed” is being developed to measure the query execution time on TCGA data set, which then has been further extended to cover the biological outcomes identified from Medline abstracts [35]. A similar platform such as FIREBROWSE^16^, Web-TCGA [36], and PCAWG^17^ have been built for TCGA dataset covering a wide range of genomic signatures and pan-cancer analysis. Gene and methylation annotation platforms such as omics4tb^18^ and Genevisible [37] help to decipher individual genes and their association annotated from TCGA. From the computational perspective, our goal is not to create yet another repository (or database), but to link the already existing ones for use in various analytical methods. We demonstrated that well-connected datasets allow to construct complex biomedical queries (e.g., listings 2-6) covering variety of genetic and biological features (cnv, gene symbol, methylation, cell cycle, protein, pathway, etc.) that can span through broad range of multiple repositories. The enrichment/linkage between COSMIC and TCGA datasets had been crucial to identify novel mutations. The approaches taken in DoCM [38], ICGC [39], and DIRECT [40] are complementary to our work in the sense that, discoveries suggested by the BIOOPENER platform are the most likely mutations/genes/pathways which can be further validated through creating links with the “well-curated” repositories (DoCM, ICGS, and DIRECT). Such validation is outside the scope of this article; however, we do plan to include “well-curated” databases in the next phase of BIOOPENER project. Similarly, we plan to extend linking with the ICGC [39] datasets that contains primary and blood samples providing further insight into the metastasis of primary tissues. Our current work covers copy number variation (CNV), genes, somatic mutation, and promotor methylation which targets highly mutated genes (on different tissues) and associated pathways. As far as we know, the work presented in this article is one of the first initiatives in discovering biomarkers and pathways for female gynecological cancer types covering five large-scale cancer and biomedical repositories.

## Discussion

As discussed above, the NGS technologies are producing a massive amount of sequencing datasets [5, 8]. A top-up of approximately 40 petabytes of genomic information every year is foreseen from a wide variety of data sources published by human genome research centers worldwide [41]. Often, these datasets are published from isolated and different sequencing facilities. In cancer genomics, description of biological and genetic entities are available in several overlapping and complementary data sources containing complex genomic features, studies, and associations of such features [17, 42]. In order to understand the tumorigenesis, it is often the case that several genetic features, diseases, medical history, etc. are studied together, therefore, one of the key challenge in cancer genomics – a cornerstone of precision medicine – is to discover gene-disease-drug data links and associations which may provide novel insight into new drug development techniques tailored specific for an individual patient (or a group of patients) targeting prevention, diagnosis and treatment of the diseases.

In cancer genomics field massive amount of data exist with complex associations. To understand these complex associations, it requires to fetch all possible *gene-disease-drug* combinations, for instance:

- Multiple pathways are involved to translate a particular gene
- A single disease can be treated by eliminating effect of the combination of multiple drugs
- Selection of these drugs is majorally based on the inhibitors (i.e., combination of *gene-pathways*)
- Effect of one pathway alteration can change the modification of single gene and yields into multiple genes

In this article, we aimed to understand the associations between genetic, cellular and molecular features across isolated cancer datasets giving insight into cancer progression from normal to diseased tissues. Correlation of genes in OV, UCS, UCEC, & CESC clearly indicates that gynecologically induced cancers do have common mechanism and overlapping pathways. Which means, a drug created for one cancer type has a higher probability to be effective for other associated cancer types.

## Conclusion

In this paper, we have presented a data-interlinked platform called BIOOPENER which enables querying different types of mutations and genomic alterations to contribute to molecular and clinical insights of cancer by defining most relevant variants and their prioritization. This knowledge could be highly advantageous for a targeted therapy and precision medicine based on gene expression data. The presented experiments are based on COSMIC, TCGA, REACTOME, KEGG, GO and HBM 2.0 datasets and have been used to identify sets of genes with relevance for four female gynecological cancer types - Ovarian (OV), Uterine Corpus Endometrial Carcinoma (UCS), Uterine Carcinosarcoma (UCEC), Cervical Squamous Cell Carcinoma and Endocervical Adenocarcinoma (UCES) - covering the 16 healthy tissue-specific genes from Illumina Human Body Map 2.0. We discovered 20 biomarkers (genes) in terms of mutation frequencies and CNV along with the promoter level changes in methylation data. We discovered three potential pathways causing promoter changes in gynecological cancers. In future, we plan to extend by covering the breast cancer type including additional genomic signatures, e.g., fusion gene, structural variations.

## Endnotes

^1^ http://www.nature.com/nature/journal/v537/n7619_supp/full/537S49a.html.

^2^ https://tcga-data.nci.nih.gov/tcga/.

^3^ http://cancer.sanger.ac.uk/cosmic.

^4^ https://www.ebi.ac.uk/gxa/experiments/E-MTAB-513.

^5^ http://bioopenerproject.insight-centre.org.

^6^ https://github.com/yasarkhangithub/BioOpener/blob/master/Top_100_Gene_List.txt.

^7^ http://bio2rdf.org/.

^8^ ftp://ftp.ebi.ac.uk/pub/databases/RDF/reactome.

^9^ http://download.bio2rdf.org/release/3/kegg/.

^10^ http://download.bio2rdf.org/release/3/goa/.

^11^ http://bio2rdf.org/.

^12^ http://www.cbioportal.org/.

^13^ https://omictools.com/cnv-annotation-category.

^14^ https://genome-cancer.ucsc.edu/.

^15^ https://github.com/yasarkhangithub/BioOpener/blob/master/Mutation_Key_Genes_Cancerwise.xlsx.

^16^ http://firebrowse.org/.

^17^ http://pancancer.info/.

^18^ http://www.omics4tb.org/.
